# IAA Synthesis Pathway of *Fitibacillus barbaricus* WL35 and Its Regulatory Gene Expression Levels in Potato (*Solanum tuberosum* L.)

**DOI:** 10.3390/microorganisms12081530

**Published:** 2024-07-26

**Authors:** Xiaoyu Li, Huan Tao, Shisong Wang, Di Zhang, Xingyao Xiong, Yanfei Cai

**Affiliations:** 1 College of Natural Resources and Environment, South China Agricultural University, Guangzhou 510642, China; xiaoyu1999@stu.scau.edu.cn (X.L.); taohuanscau@163.com (H.T.);; 2 Agricultural Genomics Institute at Shenzhen, Chinese Academy of Agricultural Sciences, Shenzhen 518124, China

**Keywords:** potato, indole-3-acetic acid, *Fitibacillus barbaricus*, promastigote, synthesis pathway, transcriptome

## Abstract

Indole-3-acetic acid (IAA), as an important regulator of potato growth, seriously affects the growth and yield of potato. Although many studies have reported that IAA-producing Bacillus can promote plant growth, little research has been conducted on its synthesis pathway and molecular mechanisms. In this study, an IAA-producing strain WL35 was identified as *Fitibacillus barbaricus*, and its yield was 48.79 mg·L^−1^. The results of the pot experiments showed that WL35 significantly increased plant height, stem thickness, chlorophyll content, and number of leaves of potato plants by 31.68%, 30.03%, 32.93%, and 36.59%, respectively. In addition, in the field experiments, WL35-treated plants increased commercial potato yield by 16.45%, vitamin C content by 16.35%, protein content by 75%, starch content by 6.60%, and the nitrogen, phosphorus, and potassium accumulation by 9.98%, 12.70%, and 26.76%, respectively. Meanwhile, the synthetic pathway of WL35 was found to be dominated by the tryptophan-dependent pathway, the IAM, TAM, and IPA pathways worked together, and the pathways that played a role at different times were different. Furthermore, RNA-seq analysis showed that there were a total of 2875 DEGs regulated in the samples treated with WL35 seed dressing compared with the CK, of which 1458 genes were up-regulated and 1417 genes were down-regulated. Potato roots express differential genes enriched in processes such as carbohydrate metabolism processes and cellular polysaccharide metabolism, which regulate potato plant growth and development. The above results provide a theoretical basis for the further exploration of the synthesis pathway of IAA and its growth-promoting mechanism in potato.

## 1. Introduction

As an important food crop and vegetable, the safe production of potato is directly related to people’s quality of life; therefore, guaranteeing the supply, quality, and safety of potato helps to maintain social stability and improve people’s livelihood [1,2]. At present, however, the main reliance is on the application of chemical fertilizers and pesticides, and the massive application of chemical fertilizers has led to the destruction of the soil and pollution of the environment, triggering a series of problems such as pesticide residues in agricultural products [3,4]. From various considerations, the development of environmentally friendly, safe, green, and sustainable microbial fertilizers that can be applied to agricultural production has become a necessary path for modern agriculture.

Plant growth-promoting rhizobacteria (PGPR) are microorganisms living in the soil around plant roots or attached to the roots [5], which can directly or indirectly promote plant growth by activating soil nutrients, improving plant inter-root nutrients [6], producing plant growth regulators [5,7,8], or antimicrobial actives [9]. There is a rich variety of inter-root biotrophic bacteria, among which the most studied ones are *Bacillus*, *Pseudomonas*, *Agrobacterium*, and so on [10,11]. Bacillus can produce spores, display very strong resistance to a variety of stresses, and are able to survive in some extreme environments [12], which promotes Bacillus products’ long shelf-life and field adaptability [13]. For example, *B. amyloliquefaciens* B9601-Y2 has the effect of promoting plant growth [14]; *B. velezensis* Y6 has the effect of promoting plant growth and increasing yield [15]. *B. velezensis* YYC could induce the expression of tomato genes related to auxin, gibberellin, jasmonic acid, and salicylic acid, which promote tomato growth [16]. Bacillus is also one of the most diverse genera of PGPR, and its growth-promoting ability and some of its mechanisms have been widely studied, with a broad potential for application.

Indole-3-acetic acid (IAA), also known as growth hormone, is a compound widely found in plants and animals and is one of the plant hormones. Within a certain concentration range, indoleacetic acid can promote cell elongation and growth and it can also stimulate the division of root progenitor cells at the base of plant branch cuttings and promote rooting. Some microorganisms can also regulate host physiological processes by synthesizing indoleacetic acid, such as the effect on the host immune system, thereby increasing protection against external stresses and, thus, promoting potato growth and yield [17]. The biosynthetic pathway of IAA involves a number of different precursors and intermediate metabolites, and these pathways can be divided into two broad categories: tryptophan-dependent and non-tryptophan-dependent. Tryptophan is the main precursor for the synthesis of IAA, and plants can convert tryptophan to IAA through several pathways, including the indole-3-pyruvic acid pathway (IPA/IpyA), the tryptamine pathway (TAM), the indole-3-acetonitrile pathway (IAN), the indole-3-acetamide pathway (IAM), and the tryptophan side-chain oxidase pathway (TSO). In addition to the tryptophan-dependent pathways mentioned above, plants can synthesize IAA through non-tryptophan-dependent pathways. These pathways are often named after specific intermediate metabolites but the specifics of these pathways are not well understood [18,19]. Current reports on the bacterial IAA synthesis pathway are mostly found in Gram-negative bacteria, with relatively few studies related to Gram-positive bacteria. In addition to regulating their physiological functions, IAA produced by microorganisms can often participate as a signaling molecule in the interaction between microorganisms and plants, regulating plant growth and development [20]. However, there are fewer studies on the mechanism of interaction between IAA-producing strains and potato, and the mechanism of their growth-promoting effects is not clear.

In this study, a strain of *Fitibacillus barbaricus* WL35 with high IAA-producing capacity was isolated from the plant inter-root soil, its growth-promoting effect on potato plants was verified by potting and field trials, and the metabolites during the growth process of WL35 were investigated to clarify the pathway of IAA synthesis. At the same time, transcriptomic analysis of the potted potato root system was studied to investigate the interaction mechanism. The results will help to better explain the growth-promoting effect of *F. barbaricus* WL35 on potato plants and provide a theoretical basis for the application of *F. barbaricus* WL35.

## 2. Materials and Methods

### 2.1. Screening of IAA-Producing Strains

Soil samples were collected from the inter-root soil of the crop at a South China farm, Guangzhou, China. A total of 10.0 g of soil samples were placed in a 250 mL triangular flask containing 90 mL of sterile water (containing 5–7 glass beads), shaken at 180 r·min^−1^ for 30 min. Then, 5 mL of the supernatant was put into a test tube and heated at 90 °C for 10 min, and then gradient dilution was carried out to 10^−5^ times. Afterward, we took 5 mL of supernatant into a test tube, heated it in a 90 °C water bath for 10 min, then made a gradient dilution to 10^−5^ times. The diluted solution was spread on the Luria–Bertani (LB) agar medium, the morphology of the single colony cultured after inverted incubation at 37 °C for 24 h was observed, and the single colonies with different morphology characteristics were selected to be numbered and then preserved by the line of the LB plate [21].

The isolated strains were subjected to an IAA qualitative test, the strains were inoculated into fresh 4 mL LB liquid medium test tubes (tryptophan content was 100 mg·L^−1^), and placed into the shaker at 30 °C, 180 r·min^−1^ to incubate for 24 h. Then, 100 μL of the culture solution was mixed with an equal amount of Salkowski’s colorimetric solution on the white ceramic plate, and left to stand for 30 min away from the light to carry out. If the color of the color reaction mixture became red, it indicated that the strain had the ability to produce IAA; strains that could produce IAA were recorded and photographed. IAA standard solution (50 mg·L^−1^) and liquid LB medium (containing 100 mg·L^−1^ L-tryptophan) were used as the positive control (CK+) and the negative control (CK-), respectively [11].

For the quantitative test on the strains retained in the qualitative test, the strains were inoculated into tryptophan-containing LB liquid medium tubes with the same culture conditions as the qualitative test for 24 h, and the OD_600_ value of the bacterial solution was determined by spectrophotometer (MV754N, Shanghai Precision Scientific Instrument Co., Shanghai, China). Then, the bacterial solution was centrifuged at 8000 r·min^−1^ for 5 min to take the supernatant, an equal volume of Salkowski’s colorimetric solution was added, mixed well, and the liquid was kept away from the light for 30 min. The color reaction was carried out, and the OD_530_ measured. At the same time, the IAA standard curve was made by preparing IAA standard solution, and the IAA content was calculated based on the constructed IAA standard curve when the OD_600_ = 1.0. The strains with IAA-producing capacity above 15 mg·L^−1^ were selected for purification, preserved in 50% glycerol, and kept at −80 °C for further study [22].

### 2.2. Identification of IAA-Producing Strains

The strains on Luria–Bertani agar medium (LB) were incubated at 37 °C for 18 h. Individual colonies were removed from the plates and placed into centrifuge tubes containing 100 μL of sterile water and vortexed for 1 min. Two universal primers were used, namely, 27F (5′-AGAGTTTGATCC-TGGTCAG-3′) and 1492R (5′-GGTTACCTTACGACTT-3′), which were amplified by PCR [23,24] (Morono et al., 2014; Yoon et al., 2017). The PCR products were purified and sequenced by Prime Tech Biotech Ltd. Sequence similarity comparisons were performed using Blastn, and some related species with 16S rRNA sequences were downloaded and compared using Mega7.0. Developmental trees were constructed using the neighbor-joining method with a bootstrap value of 1000 replicates [25].

### 2.3. Potato Pot Experiment Design

The soil used was taken from a South China farm (23°08′ N, 113°16′ E). Microbial fertilizer containing 10^8^ cfu·g^−1^ cells was made by mixing strain WL35 with 25 g of diatomaceous earth and setting aside the CK, which contained bacteria-free diatomaceous earth. Potatoes were cut into uniformly sized pieces (about 25–30 g) and mixed with CK and WL35 immediately after cutting. One potato was planted in each pot (round pots with a diameter of 34 cm and a height of 21.5 cm, each containing 10 kg of soil) to a depth of about 5–6 cm, and each treatment was replicated four times [26].

On the 45th day after seedling emergence, the plant height, stem thickness, number of leaves, and chlorophyll content of the potato plants were measured. Potato tubers were harvested 90 days after the emergence of potato seedlings and potato yield was measured for each treatment.

### 2.4. Potato Field Experiment Design

On 7 January 2023, in Zhucun, Zengcheng District, Guangzhou City, Guangdong Province (113°71′ E, 23°98′ N, 3.6 m.a.s.l.), the experiment began. After turning the ground and starting the ridge, the organic and compound fertilizer was applied in the middle of the furrow. Commercial organic fertilizer: 400 kg/mu; potato compound fertilizer (15-8-22): 100 kg/mu (Ba tian China). Three replications were set up in a completely randomized block design (RCBD) with a plot area of 15.84 m^2^, divided into three monopolies with a width of 1.2 m and a length of 4.4 m (Supplementary Figure S1). Two rows of 20 potato plants were planted at a spacing of 20 cm and a planting depth of 5–6 cm. The experiment consisted of four treatments: the control: diatomaceous earth; and diatomaceous earth containing 10^8^ cfu·g^−1^ of IAA-producing strains cells for WL35, WL41, and WL56 (isolated from maize inter-root soils). Cfu count was determined based on the enzymatic method [27]:

Number of cells·g^−1^ (cfu·g^−1^) = (number of colonies) × (dilution factor).

Moreover, 60 days after potato planting, we randomly collected 40 potato plants from the middle block of each plot. We measured the number of seedlings, plant height, stem thickness, and chlorophyll content to assess the agronomic traits of each plot, and used three plots of the same treatments to determine the mean ± standard error of the mean and compared treatments using Tukey’s test. After 90 days, the yield and quality of the potatoes were measured. Protein content was determined by the Coomassie brilliant blue G-250 colorimetric method with bovine serum albumin as the standard; vitamin C content was measured by the 2,6-dichloroindophenol titrimetric method [28]; reducing sugar content was evaluated by Dinitrosalicylic Acid Reagent method [29]; and starch content was determined based on the enzymatic method described by Khabou [30].

The yield was determined by weighing the tubers from the harvested rows and calculating the weight per hectare, and the percentage of dry weight was determined after drying the fresh sample at 105 °C for 48 h [31]. Plant material aliquot was digested in a mixture of sulfuric acid (H_2_SO_4_) and hydrogen peroxide (H_2_O_2_). The total phosphorus concentration was measured by a colorimetric assay of the P in the digest, according to the Mo-Sb colorimetric method [32,33]. Total nitrogen concentration was determined by the Kjeldahl method, whereas potassium was measured using ICP-OES [34]. The N, P, and K accumulation was calculated using data on yield and N, P, and K content.

### 2.5. Exploration of the IAA Synthesis Pathway in WL35

#### 2.5.1. Determination of Metabolites of IAA Synthesis Pathway by UPLC-MS/MS

The bacterial solution was cultured in LB medium overnight and added to 1 mmol·L^−1^ of 100 mL LB-Trp medium at an inoculation ratio of 1%; inoculated with an equal amount of LB medium as CK; incubated for 48 h at 30 °C, 180 rpm; centrifuged at 4 °C, 8000 rpm for 7 min; and the supernatant was acidified with 6 M HCl to pH = 2.5 and extracted twice with two times the volume of ethyl acetate. Then, the ethyl acetate layer was extracted; it underwent rotary evaporation at 37 °C; was re-dissolved with 10 mL of methanol (HPLC grade); filtered with 0.22 μm organic membrane into brown feeder; and then stored in a refrigerator at −20 °C for later use. The test was repeated three times.

Chromatographic-grade TAM, IAM, IAA, IAN, Indole-3-lactic acid (ILA), Tryptophol (TOL) were used as the test standards. The HPLC-grade methanol was dissolved to form a master batch of 200 mg·L^−1^, and the same volume of the master batch was mixed to form a mixture of standards; a single standard was used as a control and was stored at 4 °C. Ultra-Performance Liquid Chromatography–Tandem Mass Spectrometry (UPLC-MS/MS): An ExionLC AC liquid chromatography system coupled with an AB SCIEX triple quadrupole tandem mass spectrometer (Woodlands Central Indus. Estate, Singapore) was used for the analysis of the analytes. The analytes were operated with an electrospray ionization source (ESI) in multiple reaction monitoring (MRM) mode (Supplementary Table S1) with an electrospray voltage of 4 kV and a capillary temperature of 330 °C. The mass spectrometry was performed in the mass range of 10–300 and the data acquisition time was 21 min. The parameters of the MRM mass spectrometry are shown in Supplementary Table S1. The chromatographic column was a Bonshell C18 (2.1 × 50 mm, 2.7 μm) with a flow rate of 0.3 mL·min^−1^ and an injection volume of 1 μL, and the temperature of the column was 30 °C. The mobile phase consisted of 0.2% formic acid aqueous solution (A) and acetonitrile (B), and the gradient elution was carried out according to the gradient elution of 10% B for 0~8 min, 10~90% B for 8~16 min, 90~95% B for 16~21 min, and 10% B for 21~23 min.

#### 2.5.2. Determination of Intermediate Content of Bacteria Solution at Different Incubation Times by HPLC

The culture time of the strains was set at 16 h, 24 h, 36 h, 48 h, 60 h, and 72 h. Each treatment was repeated three times, and the concentration treatment was carried out after culture. The inoculation method, culture conditions, concentration treatment, and storage conditions were the same as those in the UPLC-MS/MS experiment. Optimization of liquid chromatographic conditions: Agilent high-performance liquid chromatography (HPLC) was used; the column was CNWAthena C18 (4.6 × 250 mm, 5 μm); the flow rate was 1 mL·min^−1^; the column temperature was 30 °C; the detection wavelength was 280 nm; and the detection time was 60 min. The mobile phases were 0.2% acetic acid aqueous solution (A) and acetonitrile (B), and the isocratic elution was performed in the ratio of 80% A, 20% B. The concentrations of TAM, ILA, IAM, IAA, TOL, and IAN mixed standards of 2.5, 5, 7.5, 10, 12.5, and 15 mL·L^−1^ were prepared using chromatographic methanol as the solvent. The standard curve was plotted by the peak area versus the concentration, and a single standard was used to determine the retention time of each standard, and the content of the metabolites in the bacterial solution was calculated by comparing it with the corresponding peak area of the bacterial solution samples.

### 2.6. qRT-PCR Analysis

The expression levels of genes in the RNA sequencing results were determined by performing quantitative real-time polymerase chain reaction (qRT-PCR) analysis of selected DEGs. The primers used were designed with Primer 5.0 (Supplementary Table S2). *F. barbaricus* WL35 was incubated in LB medium with or without tryptophan, and samples were taken at 16 h, 24 h, 36 h, 48 h, 60 h, and 72 h. The pyrG2 gene was used as the control, and the 2^−ΔΔCt^ method was adopted to calculate the relative expression of the gene to be tested. A real-time PCR system (QuantStudio 6 Flex, Applied Biosystems, Carlsbad, CA, USA) was used for qRT-PCR. The experimental procedure for qRT-PCR is described in the Supplementary Materials.

### 2.7. Sample Collection, RNA Extraction, Library Construction, and Sequencing

To evaluate the effect of WL35 on the transcriptome of potato, the following experiments were performed. Firstly, samples were collected from the tender portion of the potato root system in each of the CK and WL35 treatments in the field 45 days after potato seedlings emerged. The potato roots were washed with sterile water three times, blotted dry with sterile filter paper, wrapped in tinfoil, labeled and frozen in liquid nitrogen immediately, and then stored at −80 °C for subsequent RNA extraction. Three independent biological replicates of each sample were taken. The samples were sorted and sent to Nanjing Paisano Gene Technology for RNA extraction and transcriptome sequencing.

Total RNA was extracted with Trizol Reagent (Qing Ke Biological Co., Ltd., Beijing, China). The concentration, purity, and integrity of the RNA were then assessed using a NanoDrop spectrophotometer (Thermo Scientific, Waltham, MA, USA). Using poly-T oligo-attached magnetic beads, mRNA was first separated from total RNA. Fragmentation was carried out with divalent cations under elevated temperature in an Illumina proprietary fragmentation buffer. Illumina PE adapter oligonucleotides were ligated to prepare for hybridization after the 3′ends of the DNA fragments were adenylated. The library fragments were purified using Beckman Coulter’s AMPure XP Technology (Beverly, CA, USA) to choose cDNA fragments that were the desirable 400–500 bp in length. DNA fragments with ligated adaptor molecules on both ends were selectively enriched with Illumina PCR Primer Cocktail in a 15-cycle PCR reaction. Products were purified (AMPure XP system, Beverly, CA, USA) and quantified by use of the Agilent high-sensitivity DNA assay on a Bioanalyzer 2100 system. The sequencing library was then sequenced on NovaSeq 6000 platform (Illumina) in Shanghai Personal Biotechnology Co. Ltd., Shanghai, China.

### 2.8. Transcriptome Analysis

Samples were sequenced on the platform to produce picture files, which were then processed by the platform’s software (https://www.genescloud.cn/home accessed on 24 March 2023) to produce the original data in FASTQ format (Raw Data). We filtered the sequencing data using the Cutadapt (v1.15) software to obtain high-quality sequences (Clean Data) for further analysis. The reference genome and gene annotation files were downloaded from the genome website. The filtered reads were mapped to the reference genome using HISA T2 (v2.0.5). The differences in the gene expressions were analyzed by DESeq (1.39.0).

All the genes were mapped to Terms in the Gene Ontology (GO) database and the number of differentially enriched genes was calculated in each Term. We calculated the *p*-value using the hypergeometric distribution approach while using topGO (2.40.0) to perform GO enrichment analysis on the differential genes (the benchmark for substantial enrichment is *p*-value 0.05). Additionally, we located the GO word that is markedly enriched by differential genes to ascertain the primary biological functions carried out by differential genes. ClusterProfiler (3.16.1) software was used to carry out the enrichment analysis of the Kyoto Encyclopedia of Genes and Genomes (KEGG) pathway of differential genes, focusing on the significant enrichment pathway with *p* < 0.05.

### 2.9. Statistical Analysis

Statistical analysis was performed with Microsoft Excel 2013 and IBM SPSS Statistics 26. Duncan’s test and independent samples *t*-tests (*p* < 0.05) were used for significance testing. Origin was used to create graphs.

## 3. Results

### 3.1. The Screening and Identification of IAA-Producing Strains

**Figure 1:**
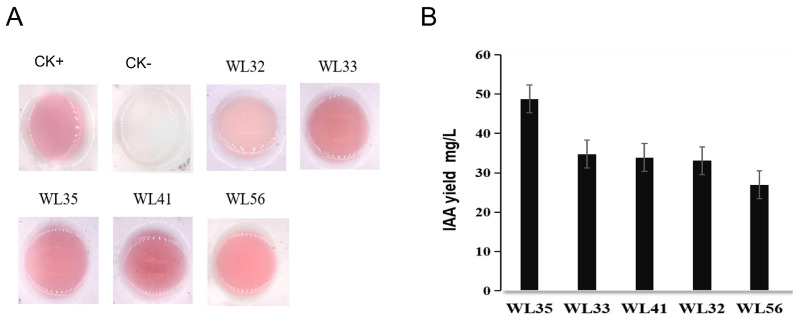
Qualitative and quantitative testing of strains. (**A**) Qualitative experimental color rendering diagram. (**B**) IAA content of different strains.

Several IAA-producing strains were isolated from the rhizosphere soil of maize (23°08′ N, 113°16′ E) at the farm of South China Agricultural University (SCAU), and the IAA-producing ability of the strains was determined qualitatively and quantitatively by Salkowski’s colorimetric assay, among which, the strains that produced higher amounts of IAA were WL35, WL41, and WL56, with the IAA yields of 48.79 mg·L^−1^, 33.91 mg·L^−1^, and 26.97 mg·L^−1^ (Figure 1). These strains were observed morphologically on LB medium after 24 h of growth. The strains were observed under the microscope as Gram-positive, spore-producing bacteria with rod-shaped cells (Supplementary Figure S2). The strains with better IAA-producing ability were preserved and subjected to further study.

By 16S *rDNA* sequencing, WL35, WL41, and WL56 were identified as *Bacillus* sp. Among them, WL35 was identified as a *Fitibacillus barbaricus* with 99% homology (Supplementary Figure S3), and WL35 16S *rDNA* sequencing was uploaded to the GenBank database to obtain the GenBank accession number of PP068887.

### 3.2. Growth Promotion and Yield-Increasing Efficacy of WL35 in Pot Experiment

**Figure 2:**
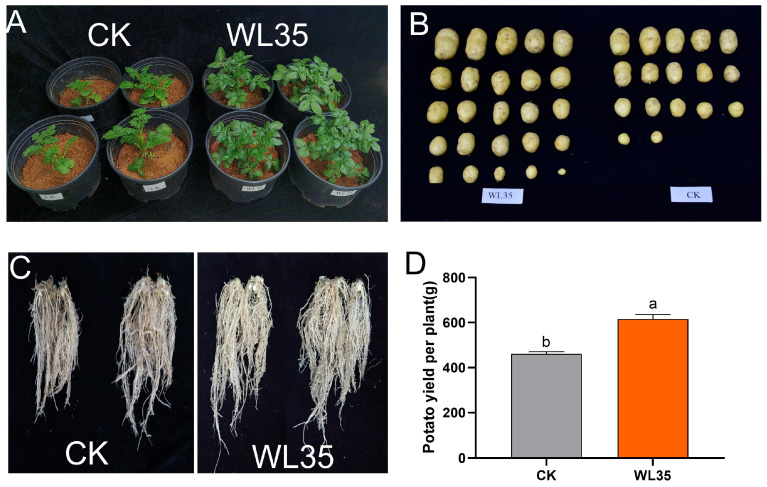
Promoting effect of WL35 on potato plants in pot experiment. (**A**) Compared with CK, WL35 significantly promoted plant growth 45 days after planting. (**B**) Compared with CK, WL35 significantly promoted plant yield 90 days after planting. (**C**) Comparative diagram of the root system. (**D**) Potato yield per plant of different treatments. a and b represent the difference significance of the results of ANOVA. Different letters indicate significant differences.

Pot experiments were conducted to determine whether WL35 promotes potato growth and increases yield. Forty-five days after the emergence of potato seedlings (Figure 2A,B), the potato plants treated with the strain WL35 seed dressing had a significant growth promotion effect relative to the CK, and the strain WL35 enhanced the plant height, stem thickness, chlorophyll content, and number of leaves of potato plants by 31.68%, 30.03%, 32.93%, and 36.59%, respectively (Table 1). Potato tubers were harvested 90 days after the emergence of potato seedlings and their yields were determined. The results showed that strain-treated pots showed a significant increase in potato yield compared to the control, with a 31.86% increase in WL35 potato weight. In addition, WL35-treated potatoes had a more compact root structure, significantly longer root length, and more fibrous roots (Figure 2C,D) (Supplementary Figures S3 and S4).

### 3.3. Growth Promotion, Yield-Increasing, and Quality Enhancement Efficacy of WL35 in Field Conditions

In order to evaluate the effect of the strain on the growth, yield, and quality of potato in the natural environment, a field experiment was conducted in this study. The results showed that treatment with microbial seed dressing made from IAA-producing strains significantly increased the seedling emergence, plant height, and chlorophyll content of potato by 14.26–28.99%, 20.58–23.93%, and 11.10–31.79% compared with the control (Supplementary Table S3).

The yield of potatoes when treated with IAA-producing strains was significantly higher, where the yield of commercial and non-commercial potatoes increased by 10.04–16.45% and 11.15–22.90%, respectively. The most effective *Fitibacillus barbaricus* WL35-treated commercial and non-commercial potatoes increased by 16.45 and 14.93%, respectively (Table 2). Meanwhile, the use of IAA-producing strains increased vitamin C content by 14.13–17.42%, protein content by 49.62–77.41%, and starch content by 0.78–6.81% (Table 3), the accumulation of N, P, and K increased by 5.26–9.98%, 4.30–12.70%, and 13.97–26.76%, respectively. Among them, WL35 increased vitamin C content by 16.35%, protein content by 75%, starch content by 6.60%, nitrogen accumulation by 9.98%, phosphorus accumulation by 12.70%, and potassium accumulation by 26.76% (Table 4).

### 3.4. Analysis of Targeted Metabolites in the IAA Synthesis Pathway

#### 3.4.1. Metabolites of IAA Synthesis Pathway in WL35

**Figure 3:**
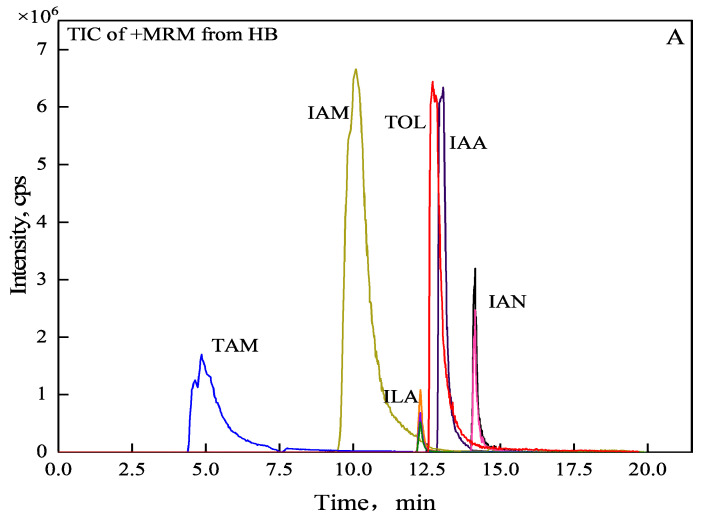

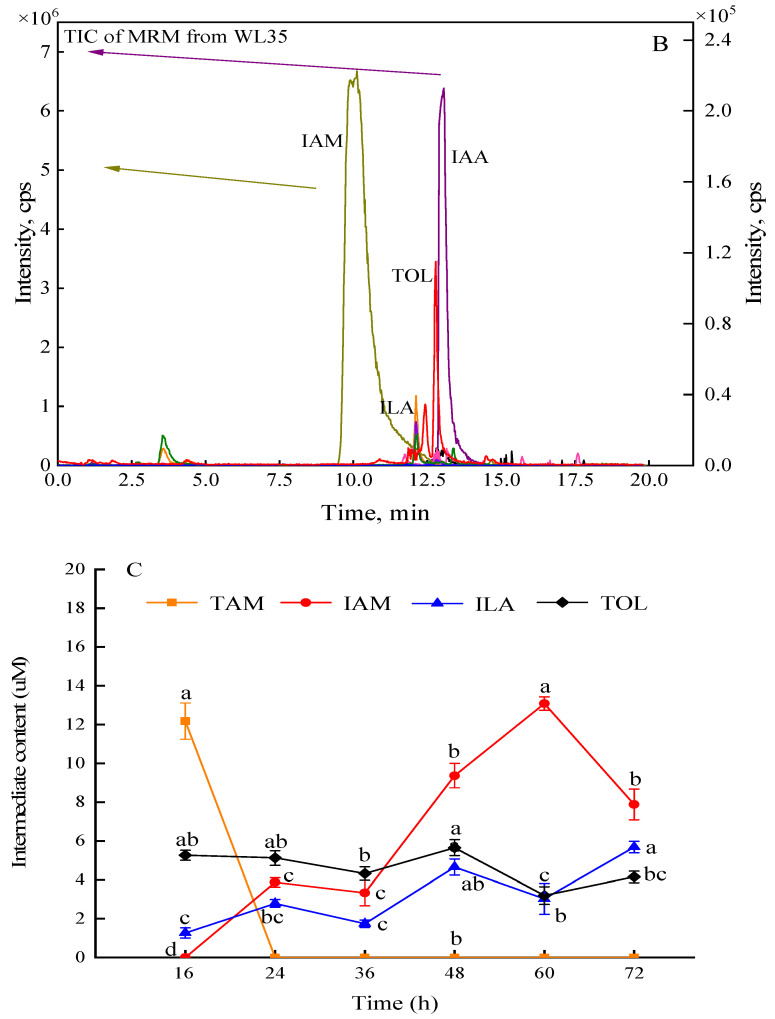
Total ion spectra of standard and WL35 in MRM mode. (**A**) Total ion spectra of mixed standards in MRM mode. (**B**) Total ion spectra of strain WL35 in MRM mode. (**C**) Changes of metabolite content over time. a, b, c, d represent the difference significance of the results of ANOVA. Different letters indicate significant differences.

Based on the results of tryptophan supplementation experiments (Supplementary Figure S6), it was initially determined that the IAA synthetic pathway of WL35 was mainly tryptophan-dependent, and six metabolites in the tryptophan-dependent synthetic pathway of IAA in the bacterial solution were further detected by UPLC-MS/MS. The results showed that, compared with the standard samples, corresponding fragmentation ion peaks of IAM, ILA, TOL, and IAA were detected in the supernatant of strain WL35, while TAM and IAN were not detected, and the retention time of each fragment ion was consistent with the corresponding standards (Figure 3A,B).

#### 3.4.2. Effect of Incubation Time on IAA Synthesis Pathway in WL35

According to the results of the kinetic determination of IAA production by the strain, after 16 h of incubation, the IAA production of WL35 reached more than 30 mg·L^−1^; therefore, the sampling was started at 16 h of incubation under the same conditions, the termination point of the sampling was at 72 h, and the metabolism products produced by the strain at different times were determined by HPLC after characterization of the detection by UPLC-MS/MS. The retention times of the standards TAM, ILA, IAM, IAA, TOL, and IAN were 4.812, 12.294, 10.053, 13.066, 12.956, and 14.145 min, respectively, and the RSDs of the retention times of the standards were 0.50–1.56%, which were less than 2%, with a high degree of separation and the peaks were good and did not tail, indicating that the chromatographic conditions were feasible. The results (Figure 3C) showed that ILA and TOL were consistently detected in the WL35 bacterial solution, and their contents ranged from 1.24 to 5.69 μM and 3.18 to 5.75 μM, respectively. TAM was only detected at 16 h with a higher content than other metabolites, and the signal disappeared after 24 h of incubation, IAM began to accumulate with an almost significant upward trend in concentration, and ILA and TOL content fluctuated within a certain range. ILA is the enzymatic reduction product of IPA, an intermediate of the IPA pathway, and TOL is the reduction product of Indole-3-acetaldehyde (IAAld), a common intermediate of IPA, TSO, and TAM pathways; therefore, it is hypothesized that the TAM pathway, IAM pathway, and IPA pathway work together to promote IAA synthesis.

### 3.5. qRT-PCR Analysis

In order to verify the IAA synthesis pathway, related genes in the pathway were analyzed based on the above results. A total of eight genes related to IAA synthesis in WL35 were identified, and quantitative primers were designed based on the sequences of these eight genes (Supplementary Table S2). qRT-PCR analysis revealed that the gene expression levels were up-regulated in WL35 grown under tryptophan treatment compared with non-tryptophan treatment, which is consistent with the results of the above-mentioned UPLC-MS/MS. (Supplementary Figure S7).

### 3.6. Analysis of Gene Transcript Levels in Potato Roots Inoculation with WL35

#### 3.6.1. Transcriptomic Analysis Using Illumina-Based RNA Sequencing

Three samples each from the two treatments (CK and WL35) were subjected to RNA sequencing (Table 5). The sequencing results showed that the Q20 values were all above 95%, while the Q30 values were all above 93%, indicating that the sequencing results had a high reliability. In addition, the total matching rate of the six samples sequenced was higher than 83%, the single matching rate was higher than 95%, the matching rate was high and stable, and the multiple matching rate was lower than 6%, indicating that the samples matched well with the selected reference genomes and that the selected reference genomes were suitable for the subsequent analysis of the reference transcriptomes.

#### 3.6.2. Biological Links between Differential Gene Expressions

**Figure 4:**
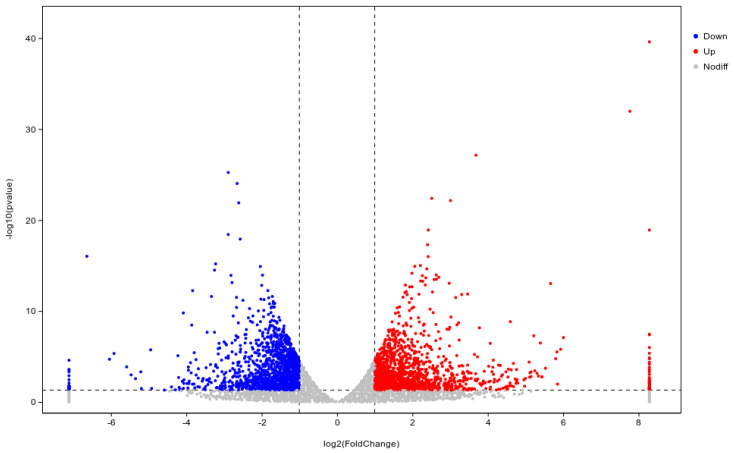
Volcano map of differentially expressed genes of CK and WL35. Red dots indicating up-regulated genes in this group; blue dots indicating down-regulated genes; and gray dots indicating non-significantly differentially expressed genes.

**Figure 5:**
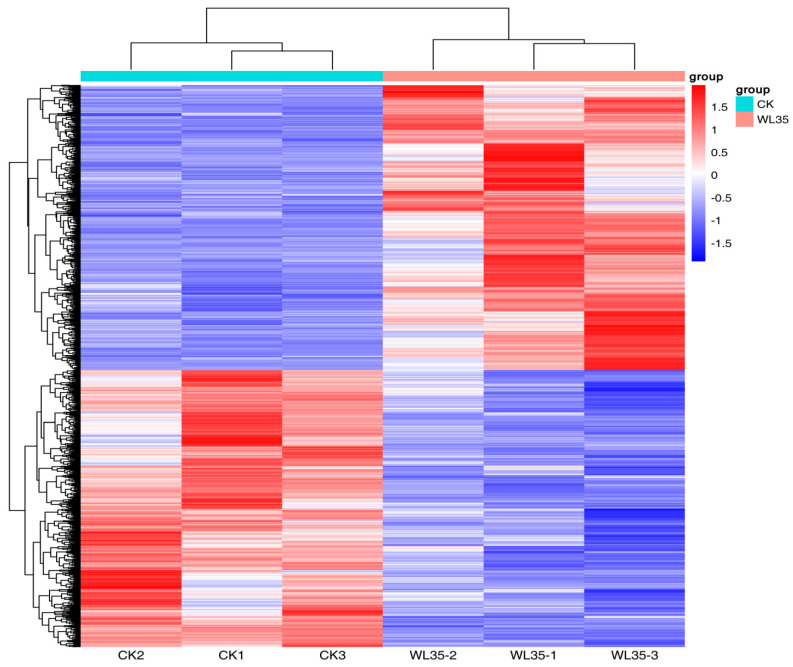
Cluster analysis of differentially expressed genes of CK and WL35. Each row represents a gene and each column represents a sample (WL35-1, WL35-2, and WL35-3 represent three replicates of WL35 sample. CK1, CK2, and CK 3 represent three replicates of CK sample). Different colors in the clustering heat map represent the different expression levels of genes. Red indicates high-expression genes and blue indicates low-expression genes, respectively.

DESeq was used to analyze gene expression differentially. A total of 2875 differentially expressed genes (DEGs) were expressed in the WL35-treated potato roots, including 1458 up-regulated genes and 1417 down-regulated genes, compared with the CK. The R ggplots2 software (https://www.genescloud.cn/home accessed on 24 March 2023) was used to draw the volcano plot of DEGs (Figure 4) to show the gene distribution, the fold difference in gene expression, and the significance results, which should be roughly symmetrical for the left and right differential gene distribution. Through expression clustering analysis (Figure 5), we found genes with unknown biological connections that are linked to each other.

#### 3.6.3. Analysis of Differential Enrichment of Gene Expression in Potato Roots

**Figure 6:**
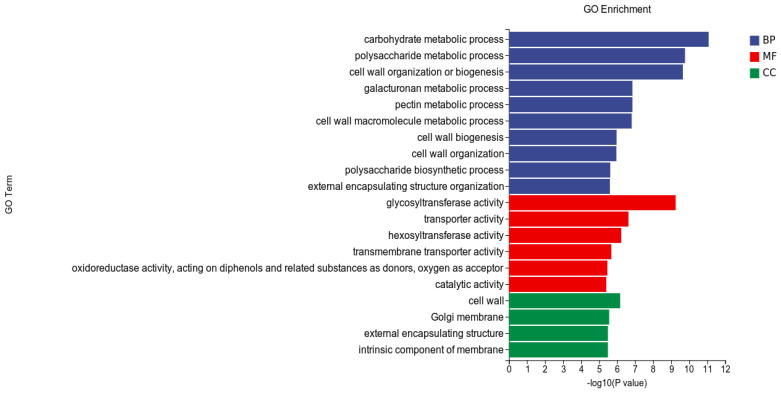
DEGs in potato tuberous of CK and WL35. The *X*-axis indicates the number and percentage of DEGs under each functional classification, the *Y*-axis represents the enriched GO functional classification.

GO analysis: In this experiment, compared with the CK, 53,932 DEGs were enriched in the GO classification of potato roots treated with WL35, with a total of 2824 functional entries, of which 16,957 DEGs were enriched in 720 functional entries in Molecular Function (MF), 9594 DEGs were enriched into 246 functional entries in Cellular Component (CC), and 27,381 DEGs were enriched into 1858 functional entries in Biological Process (BP). Among these functional entries, we performed GO enrichment analysis using topGO (https://www.genescloud.cn/home accessed on 24 March 2023) (*p* < 0.05) (Figure 6).

**Figure 7:**
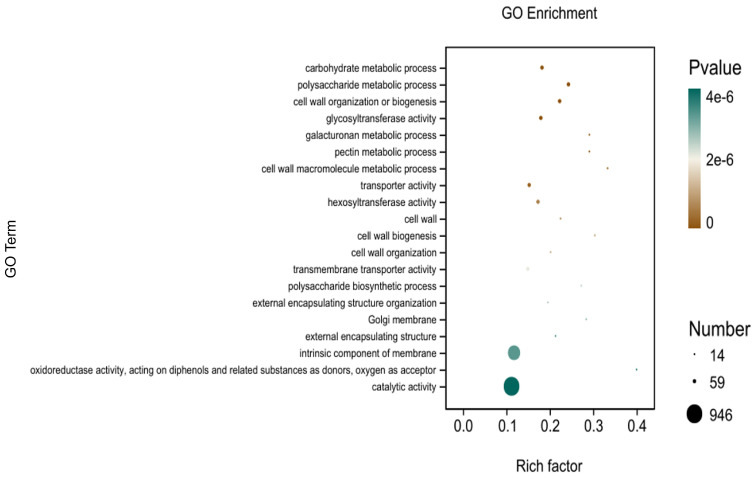
GO-enriched pathways of CK and WL35. The *X*-axis is rich factor, the *Y*-axis is GO Term name.

**Figure 8:**
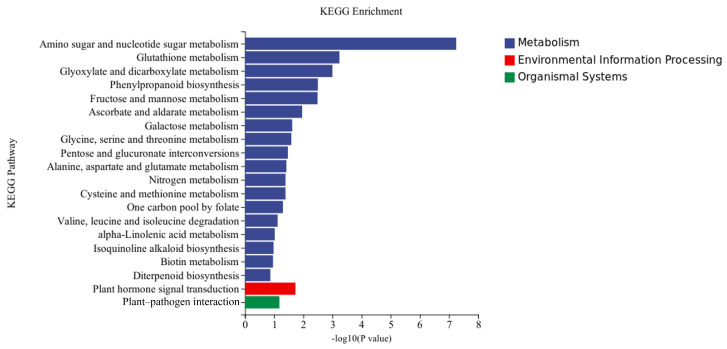
Results of KEGG enrichment analysis of CK and WL35. The *X*-axis indicates the number and percentage of DEGs under each functional classification, whereas the *Y*-axis is pathway classification.

In the three broad categories of CC, MF, and BP, the top 20 GO entries most significantly enriched were selected to be displayed in bar charts, as shown in Figure 7. Significantly differentially expressed genes in potato roots using WL35 were mainly enriched in the CC in terms of the cell wall (GO:0005618), Golgi membrane (GO:0000139), external encapsulating structure (GO:0030312), and intrinsic components of membrane (GO:0031224). In MF, it was mainly enriched in glycosyltransferase activity (GO:0016757); transporter activity (GO:0005215); hexosyltransferase activity (GO:0016758); transmembrane transporter activity (GO:0022857); oxidoreductase activity, acting on diphenols and related substances as donors and oxygen as acceptor (GO:0016682); and catalytic activity (GO:0003824). The DEGs in BP were mainly enriched in the carbohydrate metabolic process (GO:0005975), polysaccharide metabolic process (GO:0005976), cell wall organization or biogenesis (GO:0071554), galacturonan metabolic process (GO:0010393), pectin metabolic process (GO:0045488), cell wall macromolecule metabolic process (GO:0044036), cell wall biosynthesis (GO:0042546), cell wall organization (GO:0071555), polysaccharide biosynthetic process (GO:0000271), and external encapsulating structure organization (GO:0045229). In addition, in the GO factor diagram (Figure 8), DEGs were mainly distributed in the carbohydrate metabolism process (GO:0005975), polysaccharide metabolic process (GO:0005976), glycosyltransferase activity (GO:0016757), transporter activity (GO:0005215), intrinsic component of membrane (GO:0031224), and catalytic activity (GO:0003824).

**Figure 9:**
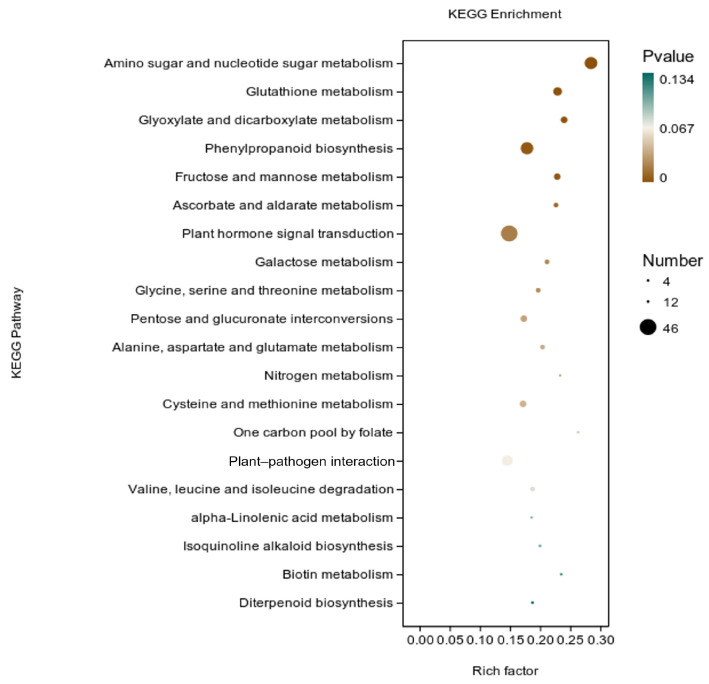
Pathways enriched in KEGG analysis of CK and WL35. The *X*-axis is rich factor, the *Y*-axis is pathway name.

KEGG analysis: KEGG analysis plots showed that 747 DEGs were annotated to 109 categorical metabolic pathways in the KEGG database for the DEGs in potato roots treated with WL35 (Figure 8). The 20 most significant pathways were mainly related to amino sugar and nucleotide sugar metabolism (sot00520); glutathione metabolism (sot00480); glyoxylate and dicarboxylate metabolism (sot00630); phenylpropanoid biosynthesis (sot00940); fructose and mannose metabolism (sot00051); ascorbate and aldarate metabolism (sot00053); galactose metabolism (sot00052); glycine, serine, and threonine metabolism (sot00260); pentose and glucuronate interconversions (sot00040); alanine, aspartate, and glutamate metabolism (sot00250); nitrogen metabolism (sot00910); cysteine and methionine metabolism (sot00270); one carbon pool by folate (sot00670); valine, leucine, and isoleucine degradation (sot00280); alpha-Linolenic acid metabolism (sot00592); Isoquinoline alkaloid biosynthesis (sot00950); biotin metabolism (sot00780); diterpenoid biosynthesis (sot00904); plant hormone signal transduction (sot04075) by environmental information processing pathways; and plant–pathogen interaction (sot04626) by organismal systems. In addition, a large number of enriched genes were distributed in plant hormone signal transduction (sot04075); amino sugar and nucleotide sugar metabolism (sot00520); glutathione metabolism (sot00480); phenylpropanoid biosynthesis (sot00940); plant–pathogen interaction (sot04626), etc. (Figure 9).

##### Strain WL35 Induces the Expression of Potato Growth-Related Genes

Brassinosteroid (BR) hormones are essential for root growth and BR biosynthesis is largely restricted to the root elongation zone [35]. Transcriptome analysis showed that potato BR biosynthesis genes were up-regulated after inoculation with strain WL35.

In the phytohormone signaling pathway, differences in the expression of endogenous hormone-related genes were observed in potato root systems compared with the control. Genes for early plant response to auxin are usually divided into three categories: Aux/IAAs, GH3, and SAURs (small auxin-up RNAs) [36]. After inoculation with the strain WL35, two genes (PGSC0003DMG400001611, PGSC0003DMG400001612) encoding SAUR family proteins in potato were up-regulated, the genes (PGSC0003DMG400026186, PGSC0003DMG400026683) encoding the GH3 family of growth hormones were up-regulated, and the gene (PGSC0003DMG400027160) encoding the DELLA proteins for growth hormone and ethylene was up-regulated. Meanwhile, the expression of the Aux/IAA3 gene (PGSC0003DMG402019457)—a transcription factor in the Aux/IAA family of genes that regulates the synthesis of endogenous IAA—and the gene (PGSC0003DMG400024997) that encodes indole-3-acetylamine synthetase were down-regulated. Six genes related to gibberellin synthesis and metabolism in the diterpene biosynthetic pathway were down-regulated, including the genes related to gibberellin 2-oxidase 1, PGSC0003DMG400021095 and PGSC0003DMG400002068; the genes related to gibberellin 2-oxidase, PGSC0003DMG400027645, PGSC0003DMG400027631, and PGSC0003DMG400027632; and the related gene of gibberellin 3-oxidase, PGSC0003DMG400016516.

##### Strain WL35 Affects the Expression of Genes Involved in Potato Metabolism

Strain WL35 caused differential expression of a number of metabolism-related genes after inoculation of potato plants. The expression of UDP-glucose 4-epimerase (UGE) related genes (PGSC0003DMG400012274, PGSC0003DMG400003054) in the amino sugar and nucleoside sugar metabolic pathways was up-regulated, and similarly, the genes (PGSC0003DMG400026854, PGSC0003DMG400026855) encoding chitin endonuclease and the HXK gene (PGSC0003DMG400000295) encoding hexokinase were up-regulated. Expression of the gene (PGSC0003DMG400013894) encoding 1-aminocyclopropane-1-carboxylic acid oxidase was up-regulated in cysteine and methionine metabolic pathways. This differential expression associated with metabolism may promote the accumulation of photosynthetic products and contribute to increased potato yields. There are 16 differential genes up-regulated in the phenylpropane biosynthesis pathway, which include the genes (PGSC0003DMG400006993, PGSC0003DMG400030382) encoding class III peroxidases (class III PERs); the CAD genes (PGSC0003DMG400015677, PGSC0003DMG401025767) encoding cinnamyl alcohol dehydrogenase; genes (PGSC0003DMG400012658, PGSC0003DMG400014003) for enzymes related to the anthocyanin biosynthesis process; and the PRDX family genes: PGSC0003DMG400011640, PGSC0003DMG400014055, PGSC0003DMG400025803, PGSC0003DMG400025478, PGSC0003DMG400024053, and PGSC0003DMG400016223.

## 4. Discussion

### 4.1. The Screening and Identification of IAA-Producing Strains

Indole-3-acetic acid, a phytohormone that plays an important regulatory role in plants, is synthesized not only by plants themselves but also by microorganisms, including bacteria and fungi [37]. It was shown that *Bacillus aryabhattai* CH07 screened from the inter-root of maize had IAA yields of 30.93 mg·L^−1^ [38]. Lin screened 22 strains of plant rhizosphere-promoting bacteria from the inter-root of Camphorus balsamifera, of which the *Pseudomonas* (*Pseudomonas* sp.) strain XZ11 and *Bacillus pumilus* XZ13 had the strongest ability to produce IAA, with yields of 46.82 and 46.19 mg·L^−1^ [39], respectively. *Bacillus aryabhattai* HL379 strain screened showed an IAA yield of 47.8 mg·L^−1^ [40]. In this study, a strain of *Fitibacillus barbaricus* WL35 with high-yielding IAA was screened from the inter-root soil of maize, and the yields were 48.79 mg·L^−1^ after 24 h of incubation in LB medium containing 100 mg·L^−1^ tryptophan, which were higher than that of the optimal *Bacillus* sp. P3-3, with 36 h of incubation, of 36.6 mg·L^−1^, and that of Y18-3 with a yield of 34.74 mg·L^−1^ [27,41].

In addition, many studies have shown that *Bacillus* sp. could synergistically promote plant growth through one or more actions [42]. However, most of the IAA-producing strains have been reported to be monofunctional and unstable. Therefore, the screening of novel multifunctional strains with IAA production ability is of great significance for the research and application of microbial fertilizers.

### 4.2. The Effects of IAA-Producing Strains on Potato Plant Growth and Yields

IAA-producing strains have a broad-spectrum growth-promoting effect, which significantly promotes seed germination and plant growth in cabbage, potato, maize, sugarcane, tobacco, and tomato [43,44]. Xing isolated one strain of *Bacillus* sp. for the treatment of pakchoi, and the fresh weight of the plant increased by 1.16% and 2.97% compared with the non-inoculated control [45]. Liu treated maize with different concentrations of *Bacillus subtilis* B9 solution and found that the growth-promoting effect on maize increased and then decreased as the multiplicity of the bacterial solution increased, and the treatment of maize and sugarcane single shoots with the same concentration found that the strains acted well in the seedling and sprouting stages of both crops [46]; this was consistent with the results of the pot planting test, indicating that the use of IAA-producing *Bacillus* sp. as a seed dressing could promote the growth of potato plants and enhance the yield of potatoes. More importantly, the application of IAA-producing strains can improve the yield and quality of commercial potatoes, such as vitamin C, protein, starch content, and elemental content, thus increasing the income of farmers. In subsequent studies, the soil microbiome will be further scrutinized and analyzed to explore the ecological impact of introducing this strain.

### 4.3. Exploration of the Synthesis Pathway of IAA in Growth-Promoting Strains

When different concentrations of tryptophan were added, the IAA production of WL35 was all significantly higher than that of the no-tryptophan condition, which indicates that tryptophan has an important effect on the strain’s IAA synthesis and that its synthetic pathway is dominated by the tryptophan-dependent type. The IAM pathway is a two-step reaction IAA synthesis pathway in which the tryptophan monooxygenase encoded by iaaM catalyzes the conversion of L-tryptophan to IAM, which is then converted to IAA by the indoleacetamide hydrolase encoded by the iaaH gene [47]. The TAM pathway involves the conversion of L-tryptophan to TAM by tryptophan decarboxylase, followed by conversion of TAM to indole acetaldehyde IAAld by amine oxidase, and finally to IAA by aldehyde dehydrogenase [48]. The IPA pathway has been characterized in a variety of bacteria, where L-tryptophan is converted by aminotransferase to IPA followed by the decarboxylase-catalyzed production of indole. In this branch, L-tryptophan is converted to IPA by aminotransferase, followed by the production of IAAld catalyzed by decarboxylase, and finally the conversion of IAAld to IAA by aldehyde dehydrogenase [49]. It is worth noting that indole pyruvic acid and indole acetaldehyde are unstable and can be easily reduced to ILA and TOL [50]. IAN can be converted directly to IAA by nitrile hydrolase or in a two-step reaction, first to IAM by nitrile hydratase or amidase and then to IAA by indoleacetamide hydrolase [51,52]. The TSO pathway, in which L-tryptophan is oxidized directly by tryptophan side-chain oxidase to IAAld and then converted to IAA, has only been detected in Pseudomonas fluorescens [53,54]. In this study, UPLC-MS/MS and HPLC were used to determine the metabolites in WL35 cultured at different times. It was hypothesized that the IAM, TAM, and IPA pathways existed in WL35. By characterizing the enzyme activity, Phi determined that *B. polymyxa* E681 possesses the IPA pathway, and the construction of mutants of the key enzyme putative gene revealed the presence of the IAN pathway in *B. amyloliquefaciens* FZB42 [55,56]. Shao identified, by a combination of chemical and genetic analyses, the presence of *B. velezensis* SQR9 candidate genes for IPA pathway-associated enzymes and validated the potential intact IPA pathway of SQR9 [57]. This is consistent with the results of this study.

### 4.4. Differential Enrichment Analysis of Gene Expression in Potato Roots

Transcriptome analysis of potato roots showed that a considerable number of genes were differentially expressed in response to inoculation with strain WL35. Strain WL35 enhances growth hormone signaling in potato. The expression of the SAUR family of protein genes, the GH3 gene family of growth hormone response genes, and the class III PER genes that regulate IAA catabolism were up-regulated, whereas the expression of the differential genes that regulate the synthesis of endogenous growth hormones was down-regulated, suggesting that there may be transport conduction of exogenous plant IAA in the root system of the potato that participates in the growth and metabolic process of the plant. The SAUR gene family, the largest of the early response gene families, influences growth hormone synthesis and transport involved in plant growth through the up-regulation of growth-promoting factors and the down-regulation of growth-inhibiting factors [58,59]. The amide synthase encoded by the Gretchen Hagen 3 (GH3) gene catalyzes the binding of phytohormones such as IAA, jasmonic acid (JA), and salicylic acid (SA) to amino acids to regulate the concentration of phytohormones, and feedback regulates the growth, development, and stress-response process of the plant [60]. The phytohormone SA, salicylic acid, acts as a signaling molecule to improve crop disease resistance by regulating an increase in the content of relevant proteins when plants are infested with pathogenic microorganisms. Salicylic acid plays a key role in plant immunity, inducing an antidisease response and protecting plants from pathogens. In addition, salicylic acid affects a variety of physiological processes in plants, including growth, development, nutrient partitioning, and source/reservoir shifts, helping plants adapt to changing environments. The phytohormone JA, jasmonic acid, belongs to the lipid class of growth regulators and is involved in the regulation of plant growth and developmental processes as well as responses to environmental factors. Jasmonic acid has diverse roles in plants, including promotion of timely anther dehiscence, regulation of seed germination, root growth, stamen development, flowering time, fruit ripening, and quality. In addition, jasmonic acid, as an important adversity-signaling substance, plays an important role in regulating a variety of plant adversity stresses. In addition, jasmonic acid, together with abscisic acid (ABA), are the two most important resistance hormones used by plants to cope with adversity, and activate different resistance signaling pathways by increasing the biosynthesis of JA-Ile and ABA, thereby enhancing plant defenses [61].

The Aux/IAA gene is a transcriptional repressor of the ARF gene, which regulates the downstream growth-hormone-regulated genes through the regulation of the ARF gene [62]. In addition, Aux/IAA genes function in a number of different phytohormone signaling pathways, such as the JA, SA [63], ethylene [64], and oleaginous steroid [65]. High expression levels of these growth hormone early response gene families promote potato growth. Moreover, brassinosteroid (BR) hormone is essential for root growth [41], and inoculation with strain WL35 resulted in up-regulation of the expression of BR-related genes in potato roots and an increase in the concentration of BR, which promoted the growth of the potato root system. Genes encoding UGE, chitinase, pectinases, class III PERs, hexokinase, and PRDX were differentially up-regulated in the transcriptome of potato interacting with WL35. These genes are all involved in the cell wall’s organization or biogenesis. UDP–glucose is the substrate for the synthesis of cellulose, the basis of synthesizing cell wall polysaccharides, and plays an important role in the interconversion of nucleotide sugars in the metabolism of cell wall polysaccharides, while UGE is a key enzyme in the conversion of UDP–glucose to the synthesis of hemicelluloses and pectins [66]. Plant chitinases play a key role in combating biotic and abiotic stresses. Class III PERs are a class of plant-specific oxidoreductases involved in many physiological processes in the plant body, possessing IAA oxidase activity, controlling the catabolism of growth hormone affecting cell growth and cell wall modification, and are also involved in lignin synthesis and the plant stress response [67]. Plant chitinases were strongly expressed when plant cells were under pathogen stress or various abiotic stresses such as plant trauma, osmotic pressure, cold, heavy metal stress, and salt [68]. Pectinases affect the regulation of cell wall mechanical stability during fruit ripening, stem lengthening, tuber yield, and root formation [69]. It has also been demonstrated that pectinesterase contributes to the plant’s defense against pathogen attack. Hexokinase regulates the utilization of stored and free sugars in plants, catalyzes the phosphorylation of hexose into the glycolytic pathway [70], provides energy and intermediate metabolites for plant physiological activities, has a key role in glucose signaling, and interacts with ethylene signaling [71]. PRDX is one of the phytoprotective enzymes that increase plant resistance [72]. The up-regulation of these genes indicates that strain WL35, as a potato seed dressing, can promote the growth of the potato cell wall, promote lignin, cellulose, and pectin synthesis, and maintain the carbon flow and respiration of starch synthesized by potato, which can help to improve the environmental resilience, yield, and quality of potato.

Furthermore, analysis of other growth hormone differentially expressed genes revealed that both gibberellin and ethylene-related signaling pathways were activated in the potato root system. According to related studies, high levels of expression of gibberellin GA2oxs genes could reduce active GA content, which reduces the plant fruit set rate, seed number, fruit quality, inhibits lateral branching development, and reduces fiber yield [73]. Down-regulation of gibberellin GA2oxs and GA3oxs gene expression in WL35-treated potato roots and down-regulation of GA2oxs gene expression in response to the ethylene precursor substance ACC [42] resulted in increased GA levels, while the up-regulation of 1-aminocyclopropane-1-carboxylic acid oxidase gene expression in the ethylene synthesis pathway promoted the synthesis of ethylene in the potato root system [74], which collectively promotes the growth and development of potato and improves potato yield. This suggests that in potato, the same gene regulatory response mechanism may exist, and IAA may act as a signaling molecule in this process to regulate the growth and development of potato plants, thereby reducing the input of exogenous IAA.

## 5. Conclusions

In this study, a highly efficient IAA-producing strain, *Fitibacillus barbaricus* WL35, was screened by Salkowski’s colorimetric assay and its IAA production was 48.79 mg·L^−1^. Application of WL35 in pots and field trials significantly increased plant biomass, increased yield, and improved quality. The analysis of the intermediates identified the tryptophan-dependent pathway as the main synthesis pathway in strain WL35, and the optimal concentration was 2.0 mM. It was initially inferred that IAA synthesis in strain WL35 was the result of the combined action of the IAM, TAM, and IPA pathways and that the pathways that acted at different times were different. In addition, the results of transcriptional analyses showed that potato root expression of differential genes was enriched in processes such as carbohydrate metabolism process and cellular polysaccharide metabolism, and the strain WL35 regulated the growth and development of potato plants by inducing the gene expression of phytohormone signaling, metabolism of amino acid sugars and nucleotide sugars, glutathione metabolism, and the phenylpropane biosynthesis process. This study helps to reveal the IAA synthesis pathway of *Fitibacillus barbaricus* WL35 and its molecular mechanism to promote potato growth and provides a theoretical basis for the application and production of WL35 to improve potato yield.

## Figures and Tables

**Table 1:** Growth conditions of potato in IAA-producing strain treatments.

Treatment	Plant Height (cm)	Stem Thickness (mm)	Chlorophyll Content (SPAD)	Number of Leaves	Root Length (cm)
CK	18.75 ± 1.30 b	7.70 ± 0.50 b	25.75 ± 1.05 b	41.00 ± 1.83 b	22.04 ± 2.17 b
WL35	24.70 ± 1.44 a	10.02 ± 0.21 a	34.23 ± 2.08 a	56.00 ± 2.94 a	25.10 ± 2.51 a
WL41	22.58 ± 0.93 ab	10.03 ± 0.37 a	32.10 ± 1.66 a	52.75 ± 2.32 a	24.65 ± 2.05 ab
WL56	24.48 ± 2.12 a	9.96 ± 0.20 a	33.90 ± 1.53 a	57.25 ± 1.25 a	24.20 ± 1.62 ab

Data are expressed as means ± standard error (*n* = 4), and indicate significant differences between different treatments at *p* < 0.05 using independent samples *t*-tests. a and b represent the difference significance of the results of ANOVA. Different letters indicate significant differences.

**Table 2:** Effect of IAA-producing strains on potato field yield.

Treatment	Commercial Potato Yield (t·ha^−1^)	First-Grade Potato Yield (t·ha^−1^)	Secondary Potato Yield (t·ha^−1^)	Non-Commercial Potato Yield (t·ha^−1^)	Potato Yield (t·ha^−1^)	Yield Increase Rate (%)
CK	35.38 ± 1.39 b	19.14 ± 0.49 b	16.24 ± 0.31 b	3.23 ± 0.33 a	38.61 ± 1.09 b	/
WL35	41.16 ± 1.31 a	21.31 ± 0.74 a	19.85 ± 0.51 a	3.71 ± 0.28 a	44.87 ± 1.31 a	16.19
WL41	39.05 ± 1.66 a	19.67 ± 1.60 b	19.38 ± 1.16 a	3.97 ± 0.27 a	43.02 ± 2.66 a	11.42
WL56	38.93 ± 1.16 a	20.11 ± 1.57 ab	18.82 ± 0.86 a	3.59 ± 0.13 a	42.52 ± 1.16 a	10.11

Commercial potatoes > 75 g, first-grade potatoes > 175 g, second-grade potatoes 75 to 175 g, and non-commercial potatoes < 75 g. Data are expressed as means ± standard error (*n* = 4). Means followed by various letter (s) within the same column are significantly different from one another (*p* ≤ 0.05).

**Table 3:** Effect of IAA-producing strains on potato quality.

Treatment	Vitamin C (mg·kg^−1^)	Protein (g·kg^−1^)	Reducing Sugar (%)	Starch (%)
CK	191.83 ± 7.73 a	7.88 ± 1.48 b	0.13 ± 0.01 a	17.87 ± 1.06 a
WL35	223.19 ± 12.11 a	13.79 ± 0.99 a	0.12 ± 0.00 a	19.05 ± 0.46 a
WL41	225.12 ± 8.87 a	13.98 ± 1.67 a	0.14 ± 0.01 a	19.08 ± 0.89 a
WL56	218.93 ± 11.89 a	11.79 ± 2.01 a	0.12 ± 0.00 a	18.01 ± 0.34 a

Data are expressed as means ± standard error (*n* = 4), and different letters indicate significant differences between different treatments at *p* < 0.05 using Duncan’s test.

**Table 4:** Effect of IAA-producing strains on the accumulation of N, P, and K in potato tubers.

Treatment	N Accumulation (kg·ha^−1^)	P Accumulation (kg·ha^−1^)	K Accumulation (kg·ha^−1^)
CK	72.24 ± 0.45 a	9.07 ± 0.45 b	142.43 ± 3.24 c
WL35	79.45 ± 0.63 a	10.21 ± 0.42 a	180.55 ± 3.13 a
WL41	76.52 ± 0.61 a	9.56 ± 0.43 ab	162.33 ± 6.90 b
WL56	77.30 ± 0.59 a	9.46 ± 0.61 b	170.40 ± 4.11 a

Data are expressed as means ± standard error (*n* = 4), and different letters indicate significant differences between different treatments at *p* < 0.05 using Duncan’s test.

**Table 5:** Summary of sequencing results and comparison results.

Sample	CK-1	CK-2	CK-3	WL35-1	WL35-2	WL35-3
Bases (G)	6.56	6.51	5.64	5.62	6.46	5.59
Q30(%)	93.68	93.85	93.55	93.69	93.76	93.73
Clean Data (G)	6.05	6.03	5.24	5.20	6.02	5.18
Clean Data (%)	92.25	92.53	92.88	92.5	93.2	92.67
Total Mapped	35,526,861 (88.69%)	33,477,679 (83.88%)	30,124,258 (86.88%)	30,039,906 (87.23%)	34,444,858 (86.42%)	28,631,000 (83.42%)
Multiple Mapped	1,246,958 (3.51%)	1,212,078 (3.62%)	1,122,133 (3.73%)	1,015,168 (3.38%)	1,228,651 (3.57%)	924,566 (3.23%)
Uniquely Mapped	34,279,903 (96.49%)	32,265,601 (96.38%)	29,002,125 (96.27%)	29,024,738 (96.62%)	33,216,207 (96.43%)	27,706,434 (96.77%)

Bases (G): the total number of bases; Q30 (%): the percentage of bases with base recognition accuracy above 99.9%; Clean Data (G): base number of high-quality sequences; Clean Data %: percentage of high-quality sequence bases in sequencing bases; Total Mapped: total sequence mapped/clean reads for the reference genome; Multiple Mapped: total number of sequences mapped to multiple locations, percentage is Multiple Mapped/Total Mapped; Uniquely Mapped: total number of sequences aligned to one position only, percentage is Uniquely Mapped/Total Mapped.

## Data Availability

The data presented in this study are deposited in the National Center for Biotechnology Information (NCBI) repository, accession number PRJNA1119397.

## Supplementary Materials

The following supporting information can be downloaded at: https://www.mdpi.com/article/10.3390/microorganisms12081530/s1, Figure S1: The experimental design (RCBD) graphics in field. Figure S2: Microscopy with Gram staining and spore staining of WL35. Strain was observed under microscopy as Gram-positive, bacteriophage-producing bacteria with rod-shaped bodies. The scale is 5 μm. Figure S3: 16S rDNA phylogenetic tree of the strains. (A) Phylogenetic tree of WL35, WL41, and WL56 based on 16S rDNA. (B) Phylogenetic tree of WL35 based on *cheA*-F1 and *cheA*-R1 gene sequences. The phylogenetic trees were constructed using a neighbor-joining method, based on 1000 replications, indicated next to the branches. Figure S4. Growth of potatoes in potting experiments. (A) Potato growth 14 days after emergence. (B) Potato growth 21 days after emergence. (C) Experimental control chart of potato emergence. Figure S5. The growth-promoting effect of strain WL35 on corn crops. Figure S6: Kinetic observation of IAA production by WL35 (A). Effect of different concentrations on IAA yield (B). Figure S7: qRT-PCR relative expression level. White bars indicate the relative gene expression level of the treatment without tryptophan as measured by qRT-PCR, and black bars indicate the relative gene expression level of WL35 treated with tryptophan. Error bars indicate the standard deviation from three biological replicates. Asterisks represent statistically significant differences (** *p* < 0.01, * *p* < 0.05), ns indicates no significant difference as analyzed using independent samples *t*-tests. Table S1: Mass spectrum parameters in MRM mode. Table S2: The primers used for real-time PCR (qRT-PCR). Table S3: Agronomic traits in potato field. Table S4. The effect of 14 days of treatment of corn by the strain to promote growth. Table S5: The effect of 21 days of treatment of corn by the strain to promote grow.
