# Supplementary material for: IAA Synthesis Pathway of Fitibacillus barbaricus WL35 and Its Regulatory Gene Expression Levels in Potato (Solanum tuberosum L.)
Source: Microorganisms. 2024 Jul 26;12(8):1530. doi: 10.3390/microorganisms12081530 (PMC11356661; doi:10.3390/microorganisms12081530)
Supplement: Supplementary file 1 [file microorganisms-12-01530-s001.zip › microorganisms-3109313-supplementary.pdf]

Materials and Methods

Corn pot experiment

Corn seeds were soaked in water for 2 h, buried in the seedling holes of the seedling trays and cultivated with substrate soil, and the soil in the seedling trays was kept moist by spraying water with a spray bottle every day, and when the corn seedlings emerged from the leaves, the seedlings with the same growth were selected and transplanted into pots, the potting soil was a mixture of substrate soil and garden soil, 1 kg of soil was added to each pot, and three corn seedlings were transplanted, and the bacteriological agent was poured onto the roots of the corn seedlings, which was the culture medium. After centrifugation to remove the culture medium and then add the resuspension of sterile water, the number of bacteria in each pot was 109cfu-kg-1, four replicates were set for each treatment, and no fungicide was applied as the control CK, and the plants were watered regularly to supply the water demand. 14 d later, the growth indexes of the maize seedlings were measured, including plant height, stem thickness, leaf length, leaf width, etc., and the samples were collected at 21 d for the determination of the whole plant indexes, including plant height, stem thickness, leaf length, leaf width, etc. The whole plant indexes were also determined. At 21 d, the samples were collected to determine the whole plant indexes, including plant height, stem thickness, and the weight of the aboveground and belowground parts.

qRT-PCR Analysis

The expression levels of genes in the RNA sequencing results were determined by performing Quantitative real-time polymerase chain reaction(qRT-PCR) analysis of selected DEGs. The primers used were designed with Primer 5.0 **(Supplementary Table S2)**.

A total of 800 ng of RNA was used to synthesize cDNA. 1μL gDNA Remover and 1μL 10 × gDNA Remover Buffer, and then added into the RNA-free tube with ice bath. The volume of RNase-free double-distilled H_2O was fixed to 10μL. The reaction mixture was gently mixed, centrifuged for 3-5s, centrifuged at 60°C for 5 min, immersed in ice bath for 2 min, and centrifuged again for 3-5s. The following reagents were added to the test tube with ice bath: 4μL of 5× RT Reaction Mix, 1.0μL of SynScriptTM III RT Enzyme Mix (SynScriptTM III cDNA Synthesis Mix, China), and fixed to 20μL use RNase-free double-distilled H_2O. The mixture was gently mixed and centrifuged for 3-5 s. The reverse transcription reaction was carried out on the PCR instrument at 25°C for 10 min, 50°C for 15 min, and 85°C for 5 min. The mixed solution was stored at -20°C. The cDNA sample was diluted 10 times as the template for on-board detection. Three technical replicates for each sample were obtained for qRT-PCR, which was run at 95°C for 1 min and then at 40 cycles each of 95°C for 10s and 60°C for 30s. *F. barbaricus* WL35 was incubated in LB medium with or without tryptophan, and samples were taken at 16 h, 24 h, 36 h, 48 h, 60 h, and 72 h. The pyrG2 gene was used as the control, and the $2^{-\Delta\Delta Ct}$ method was adopted to calculate the relative expression of the gene to be tested. A real-time PCR system (QuantStudio 6 Flex, Biosystems) was used for qRT-PCR. The experimental procedure for qRT-PCR is described in the Supplement.

Results

Strain WL35 promotes the growth of corn plants

In the experiment, the fungicides were poured onto the roots of maize seedlings at the time of transplanting to pots, and the growth data of maize plants were measured and recorded on the 14th and 21st d after transplanting. According to the measurement results, the maize plants treated with the fungicide WL35 showed an increase in plant height, stem thickness, leaf length and leaf width at 14 d compared with the blank treatment, as shown in Figure 5A. Among them, WL35 increased the plant height of maize plants by 6.32%, stem thickness by 17.95%, leaf length by 2.47% and leaf width by 11.7%, with significant differences in stem thickness and leaf width. At 21 d, leaf length, leaf width, stem thickness, fresh weight and dry weight of maize plants treated with fungicide WL35 were significantly enhanced as shown in Figure 5B.

In particular, WL35 increased leaf length by 6.67%, leaf width by 13.37%, stem thickness by 16.03%, aboveground fresh weight by 14.84% and belowground fresh weight by 12.77% in maize plants.

Not only does WL35 have a growth-promoting effect on potatoes, but in corn crops, the strain also significantly promotes plant growth and increases leaf area, thereby enhancing photosynthesis. In addition, the strain also has a significant yield-enhancing effect on rice, which can significantly increase the number of tillers and thousand-grain weight of the plant, thus increasing the local income.

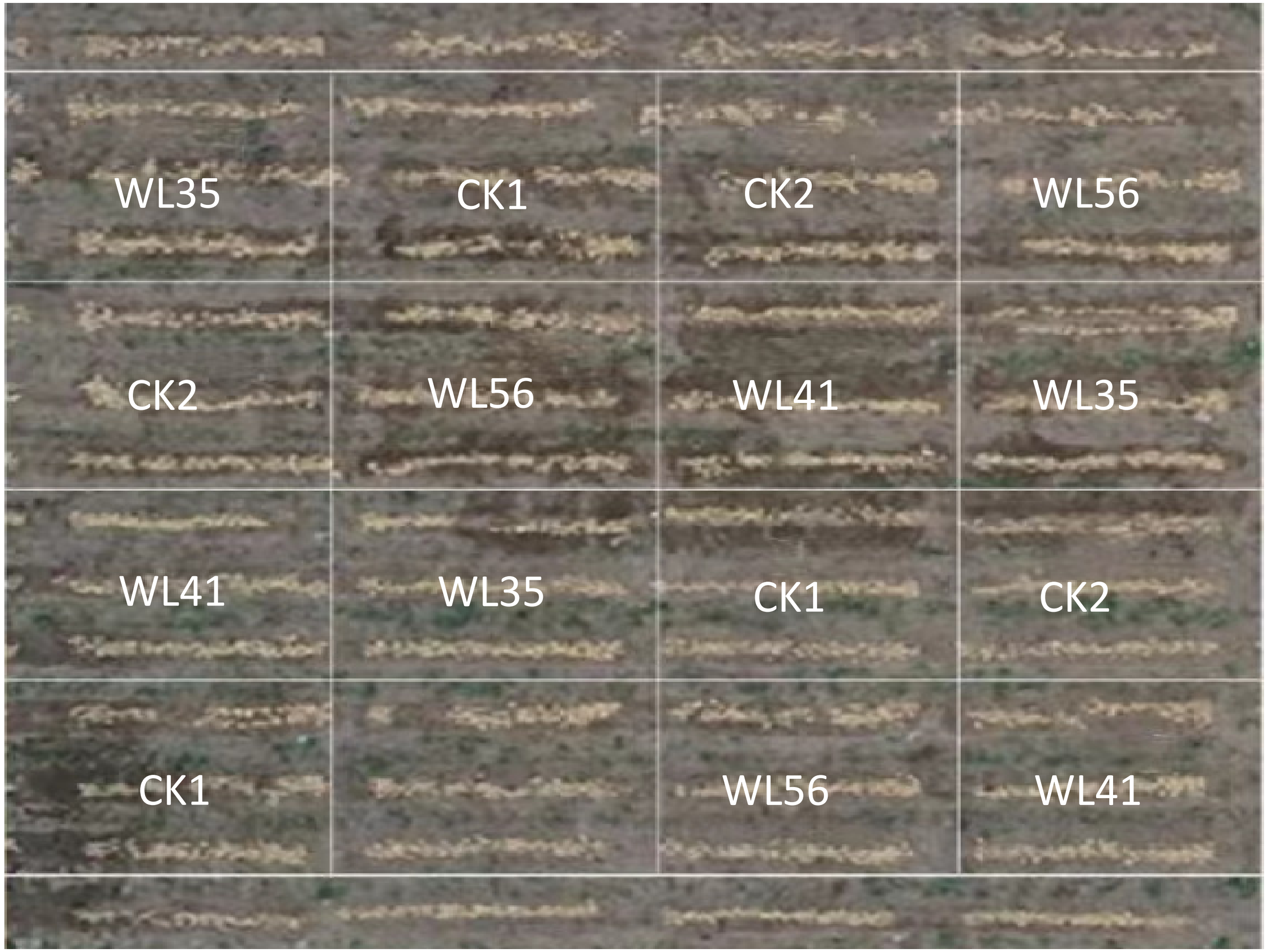

FIGURE S1: The experimental design (RCBD) graphics in field.

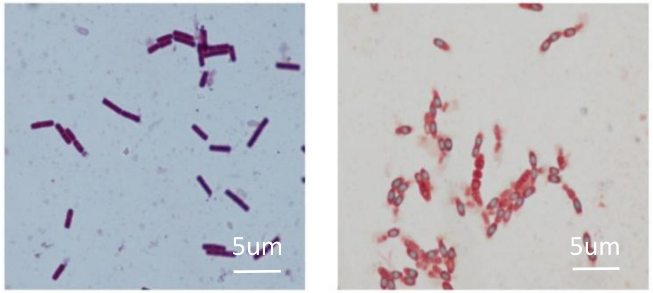

FIGURE S2. Microscopy with Gram staining and spore staining of WL35. Strain was observed under microscopy as gram-positive, bacteriophage-producing bacteria with rod-shaped bodies.The scale is 5um.

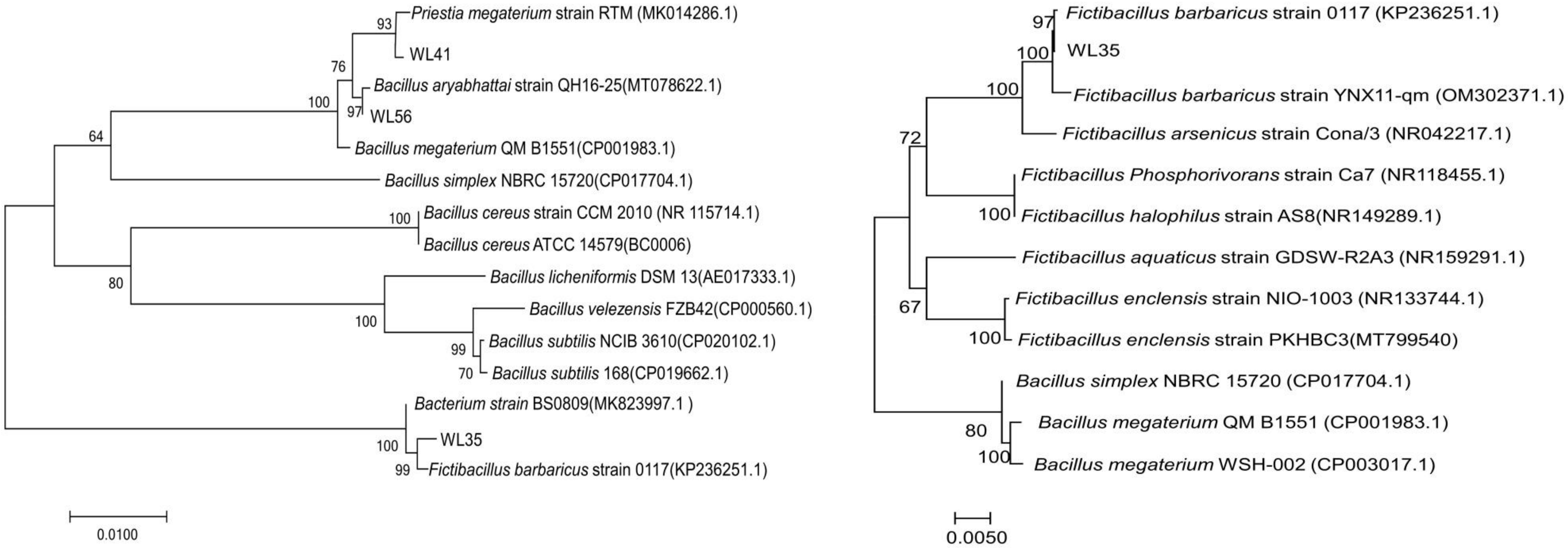

FIGURE S3. 16S rDNA phylogenetic tree of the strains. (A) Phylogenetic tree of WL35, WL41 and WL56 based on 16S rDNA. (B) Phylogenetic tree of WL35 based on *cheA*-F1 and *cheA*-R1 gene sequences. The phylogenetic trees were constructed using a neighbor-joining method. Based on 1000 replications, are indicated next to the branches.

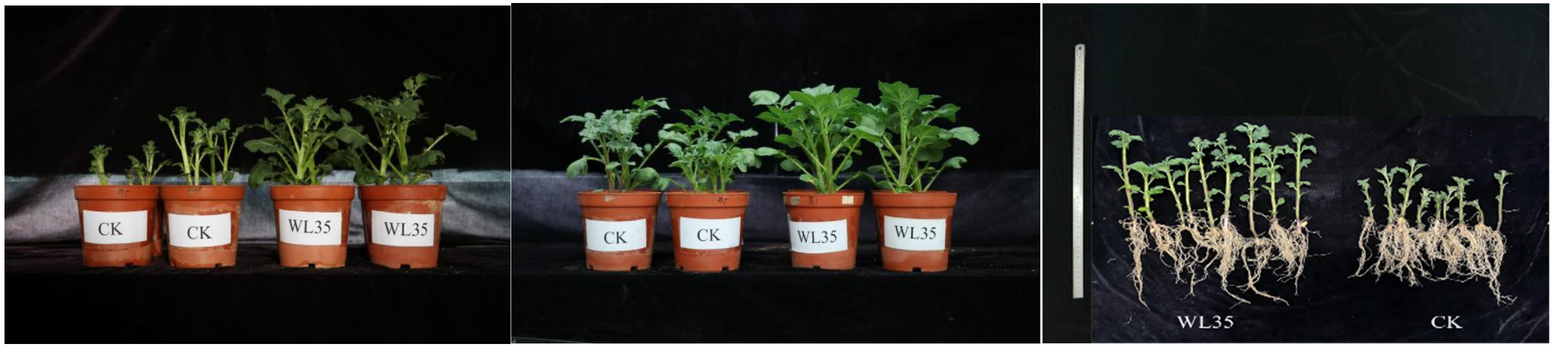

FIGURE S4. Growth of potatoes in potting experiments. (A) Potato growth at 14 days of emergence. (B) Potato growth at 21 days of emergence. (C)Experimental control chart of potato emergence.

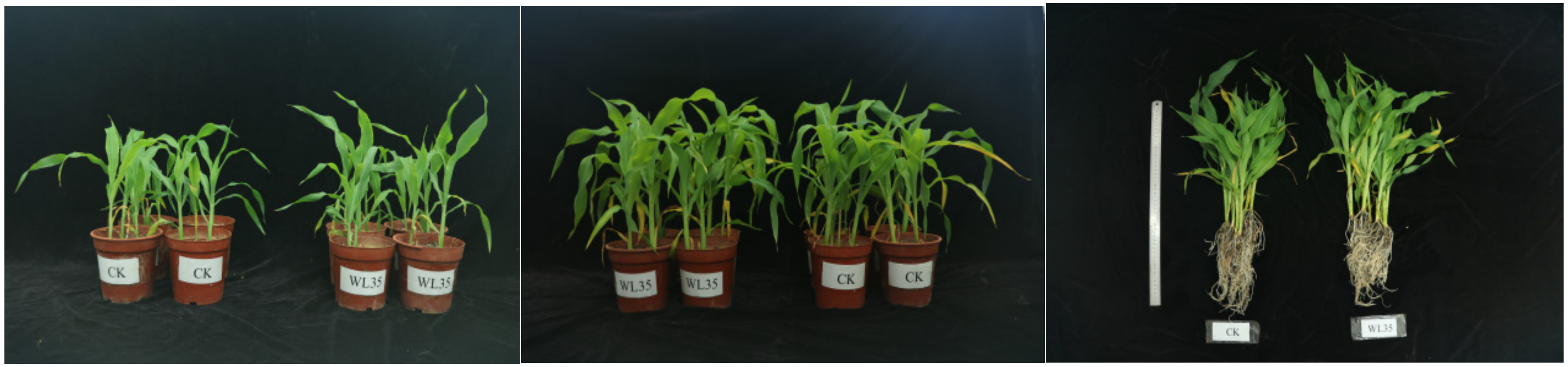

FIGURE S5. The growth-promoting effect of strain WL35 on corn crops.

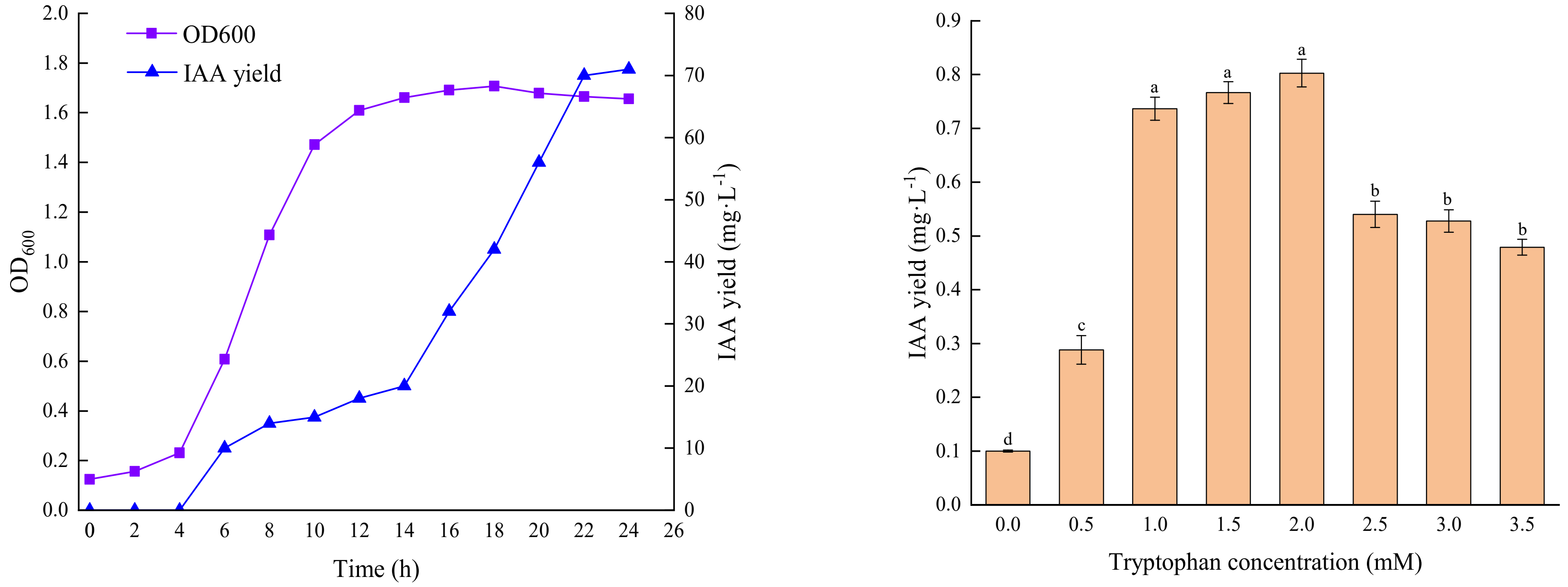

FIGURE S6:Kinetic observation of IAA production by WL35(A). Effect of different concentrations on IAA yield(B).

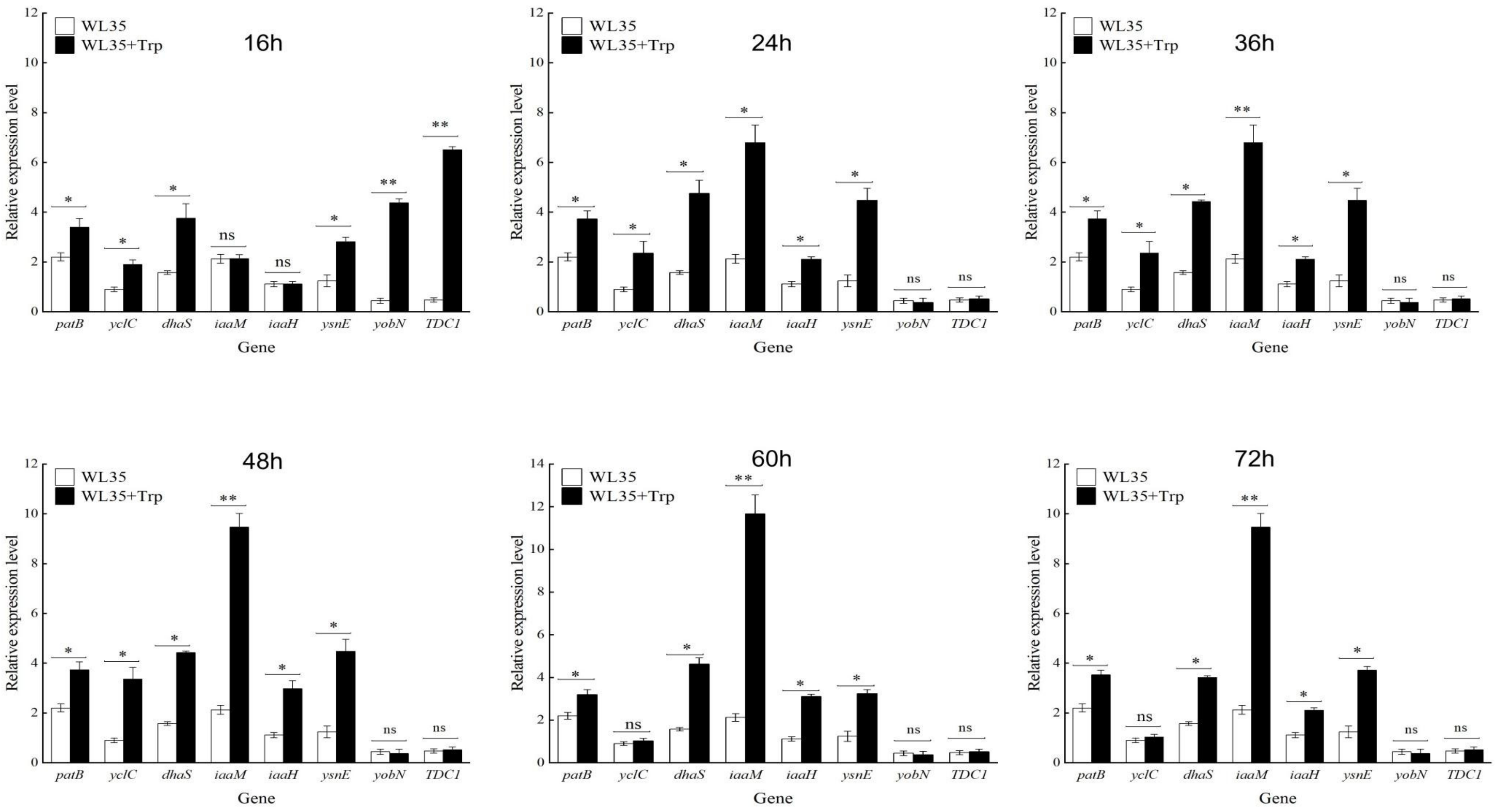

FIGURE S7: qRT-PCR relative expression level. White bars indicate the relative gene expression level of the treatment without tryptophan as measured by qRT-PCR, and black bars indicate the relative gene expression level of WL35 treated with tryptophan. Error bars indicate the standard deviation from three biological replicates. Asterisks represent statistically significant differences (**$P < 0.01$, *$P < 0.05$), ns indicates no significant difference as analyzed using Independent-samples's *t*-test.

TABLE S1. Mass spectrum parameters in MRM monitoring mode.

Compound	Parent ion (m/z)	Daughter ion (m/z)	Declustering potential (V)	Collision energy (eV)	Retention time (min)
IAA	176.0	130.0	62	18	13.06 ± 0.10
IAM	175.1	130.3	64	18	10.05 ± 0.10
	175.1	158.0	64	18	
TAM	161.0	143.6	46	15	4.81 ± 0.15
TOL	162.1	144.0	68	19	12.95 ± 0.10
ILA	206.1	118.0	60	23	12.29 ± 0.10
	206.1	130.0	60	23	
	206.1	160.2	60	23	
IAN	157.2	117.0	63	23	14.15 ± 0.10
	157.2	130.0	63	23	

TABLE S2. The primers used for real-time PCR (qRT-PCR).

Selected Genes	Gene name	Forward primer(5'-3')	Reverse primer(5'-3')
Control	pyrG2	AGTCACCCTCCTCAAACTCG	TCGTGAAGTTGTTGGCCTTG
Gene 1	patB	CAGGCACGGCTGGAAGGTA	AACTGGAGGCTGAACAACTA
Gene 2	yclC	GGTACCGAGGAATCTGATGG	CAGTTATTGATTCATTAAGTC
Gene 3	dhaS	TTAGATAACGGAAAGCCAATC	CCCAGCCAGCATAATAGCG
Gene 4	iaaM	TGAAGATGCCTGAAACGGCT	CCAGTAGCCAGGCACAAATTC
Gene 5	iaaH	CAAGAAAACACGTGGTGCAT	CGAATCCAGCTGATGTGGC
Gene 6	ysnE	AGTAACTAAACTAGAGTGGCA	CGACGCAGTCGGACTGCAA

Gene 7	yobN	TGGTTCACGAAAAGCACAGG	TACCGGAAAACTCGGTGTCAT
Gene 8	TDC	GTAAGGTATAGTATCATC	GTGAGTTAGCTAGGTTGGTGTG

TABLE S3. Agronomic traits in potato field.

Treatment	Number of seedlings	Plant height (cm)	stem thickness (mm)	chlorophyll content（SPAD）
CK	22.56±1.40c	38.77±4.82b	11.21±2.08a	27.21±1.94c
WL35	29.11±0.81a	47.95±3.91a	11.48±2.45a	35.85±1.09a
WL41	25.78±0.91b	48.04±4.64a	10.89±1.65a	33.05±1.18ab
WL56	27.44±1.12ab	46.75±5.31ab	11.91±1.79a	30.23±1.17b

Data are expressed as means ± standard error (n = 4), and different letters indicate significant differences between different treatments at $p < 0.05$ in Duncan's test.

TABLE S4. The effect of 14 days of treatment of corn by the strain to promote growth.

Treatment	Plant height (cm)	stem thickness (mm)	length of a leaf(cm)	Leaf wide(cm)
CK	31.02±2.16b	3.51±0.33b	23.88±3.73b	2.56±0.29b
WL35	32.98±4.59b	4.14±0.80a	24.47±2.84b	2.86±0.39ab
WL41	37.16±4.87a	4.41±0.56a	30.79±3.61a	3.06±0.52a
WL56	39.29±6.99a	4.16±0.60a	31.53±5.45a	2.89±0.40ab

TABLE S5. The effect of 21 days of treatment of corn by the strain to promote grow

Treatment	length of a leaf(cm)	Leaf wide(cm)	stem thickness (mm)	Fresh weight on the ground section（g）	Fresh weight in the lower ground（g）
CK	30.56±5.70c	3.29±0.37b	4.18±0.28b	9.30±2.41b	4.23±0.28b
WL35	32.56±2.10bc	3.73±0.49a	4.85±0.37a	10.68±1.49a	4.77±0.37a
WL41	36.36±3.99ab	4.07±0.53a	4.86±0.27a	10.56±1.30a	4.89±0.28a
WL56	39.29±5.34a	4.13±0.56a	4.94±0.51a	11.54±1.88a	5.28±0.51a
